# Buddleoside-Rich *Chrysanthemum indicum* L. Extract has a Beneficial Effect on Metabolic Hypertensive Rats by Inhibiting the Enteric-Origin LPS/TLR4 Pathway

**DOI:** 10.3389/fphar.2021.755140

**Published:** 2021-10-08

**Authors:** Ya-Jun Wang, Jie Su, Jing-Jing Yu, Mei-Qiu Yan, Meng-Lin Shi, Qi-Di Huang, Bo Li, Wen-Yan Wu, Rong-Shuang Xia, Si-Fan Li, Su-Hong Chen, Gui-Yuan Lv

**Affiliations:** ^1^ School of Pharmacy, Zhejiang Chinese Medical University, Hangzhou, China; ^2^ Collaborative Innovation Center of Yangtze River Delta Region Green Pharmaceuticals, Zhejiang University of Technology, Hangzhou, China

**Keywords:** metabolic hypertensive, buddleoside, lipopolysaccharide, intestinal flora, enteric-origin LPS/TLR4

## Abstract

As the number of patients with metabolic hypertension (MH) is increasing, there is an essential require for global measures to prevent and treat MH. Flavonoids such as buddleoside (BUD) from *Chrysanthemum indicum* L. are the main pharmacological components of cardiovascular activities. Previous studies have suggested that the buddleoside-rich *Chrysanthemum indicum* L. extract (BUDE) can reduce blood pressure in spontaneously hypertensive rats (SHR). However, its effect on MH and how it works remains to be researched. In this study, it was observed that BUDE could lower blood pressure, improve dyslipidemia, and decrease the level of plasma LPS in MH rats. Moreover, BUDE improved intestinal flora and increased the expression of occludin and claudin-1 in the colon, and improved the pathological injury of the colon. Western bolt and qRT-PCR experiments showed that BUDE could down-regulate TLR4 and MyD88 protein and mRNA expression and inhibit phosphorylation of IKKβ, IκBα and NF-κB p65 in vessels of MH rats. These results showed that BUDE could regulate intestinal flora, improve intestinal barrier function, reduce the production and penetration of LPS, thereby inhibiting the vascular TLR4/MyD88 pathway, improving vascular endothelial function, and ultimately lowering blood pressure in MH rats. This study provides a new mechanism of BUDE against MH by inhibiting the enteric-origin LPS/TLR4 pathway.

## Introduction

Hypertension is one of the common cardiovascular diseases. In 2015, the number of adults with hypertension worldwide reached 1.13 billion (NCD-RISC, 2017). According to the 2017 American College of Cardiology/American Heart Association (ACC/AHA) Hypertension Guidelines, hypertension is redefined as continuous systolic blood pressure ≥130 mmHg or diastolic blood pressure ≥80 mmHg (Wang Z. et al., 2018). Applying this standard, the prevalence of hypertension would increase from 32 to 45% in the United States and from 23.2 to 46.4% in China (Mills et al., 2020). Epidemiological studies have clearly established the importance of unhealthy diets, such as diets high in fat, sugar and salt, as risk factors for increased blood pressure (Shimbo, 2016). In addition, surveys suggest that consequences of hypertension are complicated by the frequent presence of comorbidities. Metabolic disorders are especially common, occurring in approximately 71.8% of patients with hypertension. Specifically, 55.3% of hypertensive patients also have abnormal blood glucose levels, and 69.5% have abnormal blood lipid profiles. Accordingly, only 10–20% of patients with simple hypertension have no symptoms other than hypertension (Zhu et al., 2013).

Thus, scholars have developed the concept of metabolic hypertension (MH) and have shown a clear causal relationship between metabolic abnormalities and hypertension (Pool, 1993; Zhu et al., 2013). The accompaniment of increased blood pressure by a variety of metabolic disorders increases cardiovascular risk and makes it difficult to achieve a good prognosis with even the most advanced treatment. Therefore, the prevention and treatment of MH will become an important focus of hypertension treatment. Unfortunately, pathophysiological mechanisms underlying MH are complex, inhibiting the development of targeted treatments. MH is known to be associated with unhealthy dietary behaviors, such as the indulgence in over-consumption of alcohol, high sugar and fat diets (ACHSFDs), as these behaviors correlate with the occurrence and development of hypertension (Zhu et al., 2016; Li et al., 2021). Importantly recent studies have identified the significance of the gastrointestinal tract, as the initial barrier for the entry of exogenous substances such as alcohol and fat into the body, in links between diet and MH (Zhu et al., 2016; Li et al., 2021). The gastrointestinal tract is not only the location of digestion and absorption, but it is also involved in the regulation of cardiovascular metabolism, and it thus may be the organ of initiation of MH (Zhu et al., 2016).

When regarding the gastrointestinal tract, attention must be paid to intestinal microorganisms, which comprise a large biome that interacts with the host and influences metabolism, immunity and inflammation (Leclercq et al., 2017). Gut microbial metabolites, such as lipopolysaccharide (LPS) and short-chain fatty acids, affect cardiovascular health (Kutryb-Zajac et al., 2019). LPS is a main component of the cell wall of Gram-negative bacilli and is a significant factor in inducing inflammation and metabolic diseases such as MH. Elevated LPS levels have been found in patients with hypertension and diabetes and in various animal models (Raybould, 2012; Kim et al., 2018; Lei et al., 2019). LPS is released upon the death of intestinal bacteria, and, typically, only a small amount of LPS is absorbed through the intestine and then cleared after entering the liver (Guerville and Boudry, 2016). When balance of intestinal flora is upset, however, LPS may be over produced, or the intestinal barrier may be damaged, leading to increased penetration of LPS that can exceed the processing capabilities of the liver. Under these circumstances, intestinal LPS can enter the systemic circulation, leading to chronic low-grade inflammation and metabolic diseases, including MH. Research suggests that diets high in fat and sugar (Parada venegas et al., 2019; Jamar et al., 2021) or alcohol consumption (Wang et al., 2014; Han et al., 2016; Leclercq et al., 2017) affect the balance of the intestinal flora, destroy the intestinal mucosal barrier, and ultimately lead to increases of LPS in the systemic circulation.

A cascade effect resulting from the vascular endothelial dysfunction induced by LPS has been reported to play a pivotal role in hypertension (Grylls et al., 2021). Studies have shown that LPS is a strong activator of vascular endothelial cells, and this activation induces endothelial dysfunction *in vivo* and *in vitro* (Kutryb-Zajac et al., 2019; Liu et al., 2020). Toll-like receptor 4 (TLR4) is highly expressed in vascular endothelium and mediates a response to LPS by activating signal transduction through myeloid differentiation factor 88 (MyD88) (Kawasaki and Kawai, 2014), a toll/interleukin-1 receptor homology domain-containing protein. In this pathway, phosphorylation of the IκB kinase (IKK) complex triggers phosphorylation of the NF-κB inhibitor IκBα. Degradation of IκBα leads to the translocation of NF-κB to the nucleus, which induces the expression of proinflammatory cytokines such as tumor necrosis factor-α (TNF-α) and interleukin-6 (IL-6), leading to vascular endothelial injury and elevated blood pressure (Xu et al., 2017). This pathway potentially liking changes in intestinal flora and intestinal pathological damage to hypertension through LPS and TLR4 suggests potential strategies to improve MH.

Importantly, preliminary studies in our laboratory have identified a flavonoid from *Chrysanthemum indicum* L., a traditional Chinese edible and medicinal plant that is a candidate inhibitor of this key pathway. Flavonoids, polysaccharides, saponins and other compounds in traditional Chinese medicine have been shown to act as prebiotics to promote the growth of some intestinal microbial species (Feng et al., 2019), and extracts from *Chrysanthemum indicum* L. have been shown to exhibit anti-inflammatory, anti-bacterial, anti-oxidant and anti-hypertensive effects, in part due to an abundance of flavonoids (Cheng et al., 2005; Zhu et al., 2005; Yu and Yu, 2007; He et al., 2012; Lv et al., 2013). Buddleoside (BUD) is one of the rich flavonoids, which exhibits anti-inflammatory and antioxidant (Xu et al., 2017; Xie et al., 2020). Our previous study found that buddleoside-rich *Chrysanthemum indicum* L. extract (BUDE) isolated by solid-liquid extraction method could lower the blood pressure of spontaneously hypertensive rats (SHR) (Yin et al., 2014), and inhibit LPS-induced vascular endothelial cell damage *in vitro* (Xu et al., 2016). Therefore, we hypothesized that the BUDE could lower the blood pressure of MH rats by inhibiting endothelial injury; however, the effects and mechanism of action of BUDE on MH rats have not been reported.

To test this hypothesis, then, Sprague Dawley rats were used to establish a rat model of MH through administration of an ACHSFD diet, which simulates the unhealthy eating habits of many humans. The success of the development of this model was assessed *via* blood pressure and serum lipid levels. After administration of BUDE, blood pressure, serum lipids, histopathological changes in liver and intestine, ultrastructural and pathological changes in blood vessels, and alterations of intestinal flora were evaluated. In this study, we demonstrated that BUDE can improve blood pressure and other markers of MH in a rat model, and we found that underlying mechanism of action may be related to the regulation of vascular endothelial function by the enteric-origin LPS/TLR4 pathway.

## Materials and Methods

### Chemicals and Reagents

*Chrysanthemum indicum* L. was purchased from Zhejiang Chinese Medical University, Traditional Chinese Medicine Decoction Pieces Co., Ltd. (Hangzhou, Zhejiang, China). BUD standard was purchased from National Institutes for Food and Drug Control, and the content of BUD was calculated as 96.6%. The standard reagents of total cholesterol (TC), triglyceride (TG), low density lipoprotein cholesterol (LDL-c), high density lipoprotein cholesterol (HDL-c), alanine aminotransferase (ALT), and aspartate aminotransferase (AST) were purchased from Ningbo Medical System Biotechnology Co., LTD (Ningbo, Zhejiang, China). Nitric oxide (NO), endothelin 1 (ET-1), endothelial nitric oxide synthase (eNOS), d-lactic acid (D-LA), tumor necrosis factor-α (TNF-α), Interleukin- 6 (IL-6) and lipopolysaccharide (LPS) kits were purchased from Jiangsu Enzyme Industrial Co., Ltd (Yancheng, Jiangsu, China). Hematoxylin and DAB colorimetry kit were purchased from Nanjing Jiancheng Institute of Biological Engineering (Nanjing, Jiangsu, China), and Eosin Y from Sigma (St.Louis, MO, United States). Occludin antibodies were purchased from Gene Tex (Alton Pkwy Irvine, CA, United States), and Claudin-1, p-IKKβ, and GAPDH antibodies were purchased from Abcam (Cambridge, UK). TLR4, IKKβ, p-IκBα, IκBα, p-NF-κB p65, NF-κB p65, *β*-actin antibodies were purchased from Cell Signaling Technology (Danvers, MA, United States). MyD88 antibody was purchased from Immunoway (Beijing, China). Trizol ^®^ Plus RNA Purification Kit, SuperScript™ III First-Strand Synthesis Supermix for qRT-PCR purchased from Invitrogen (Carlsbad, CA, United States), Rnase-free DNase Set was purchased from QIAGEN (Hilden, Germany) and Power SYBR^®^ Green PCR Master Mix from Applied Biosystems (Foster City, CA, United States).

### Preparation and Purification of BUDE

**FIGURE 1:**
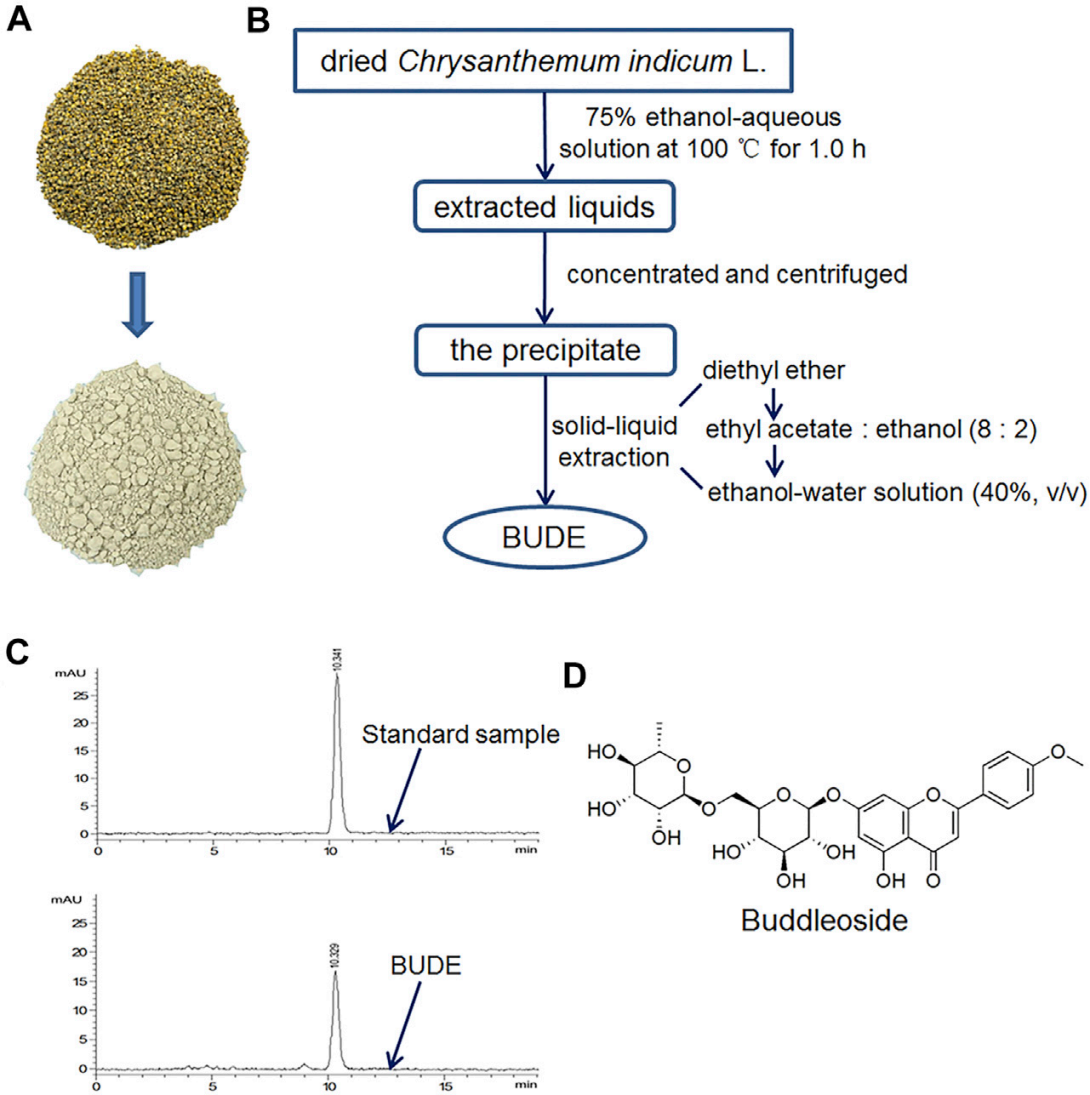
Characteristics and extraction flow chart of buddleoside-rich *Chrysanthemum* indicum L. extract (BUDE). **(A)** The representative photos of *Chrysanthemum* indicum L. and BUDE. **(B)** Extraction flowchart of BUDE **(C)** HPLC chromatograms of buddleoside (BUD) and BUDE. **(D)** The chemical structure of BUD.

BUDE was isolated and purified by the solid-liquid extraction method established in our laboratory (Yin et al., 2014). Briefly, 2.5 kg dried *Chrysanthemum indicum* L. was extracted in a Chinese medicinal extracting machine (YF-40, Beijing Donghuayuan Medical instrument Co., Ltd.) at 100°C for 1 h with 10 volumes of a 75% ethanol-water solution. This extraction was repeated three times, and the medicinal solutions were combined. The flavonoids were purified by solid-liquid extraction, through the use of four different polar solvents, which were diethyl ether, ethyl acetate, ethanol, and ethanol-water solution. After vacuum drying, BUDE dry powder was obtained, and the yield was approximately 6%. The appearance of *Chrysanthemum indicum* L. and BUDE is shown in Figure 1A. The extraction process is shown in Figure 1B.

### Analysis of BUD Content of BUDE

The content of BUD in BUDE samples was determined by analysis with an HPLC (Agilent Technologies 1,200 series) equipped with a diode array detector. HPLC chromatograms are shown in Figure 1C. A reversed phase column, Eclipse XDB-C18 (250 × 4.6 mm, 5 μm), was used. Methanol-water (52:48) was prepared as the mobile phase. The diode array detector wavelength was 334 nm. The injection volume was 10 μl. The flow rate was 1.0 ml/min. The equation of the BUD standard curve obtained with this method was y = 792.81x + 1.8054 (*R* ^2^ = 0.9992). The results of HPLC analyses demonstrated that BUDE contained 69.62 ± 0.78% of BUD. The chemical structure of BUD is shown in Figure 1D.

### Animals and Experimental Design

Fifty male Sprague-Dawley rats, weighing 180–220 g, were purchased from the Animal Supply Center of Zhejiang Academy of Medical Sciences (SCXK 2019-0002, Hangzhou). All animal experiments involved in this experiment meet ethical and legal requirements (ZSLL-2017-185).

All animals were allowed to eat standard food and drink water freely for 1 week. Then nine rats were randomly selected as a normal control group (NG) and were fed with a normal standard diet, and the remaining rats were administered ACHSFD in order to induce the MH model. The high sugar and fat diet was obtained from TROPHIC Animal Feed High-Tech Co. Ltd. (Nantong, Jiangsu, China). The diet was supplemented with 20% sucrose, 15% lard, 0.8% cholesterol, 0.2% sodium cholate and 64% standard diet. The corresponding concentration of alcohol was made from 52% alcohol (Red Star Company, Beijing, China) diluted with distilled water. In the first week, the administered alcohol concentration was gradually increased from 5 to 10% and then increased by 2% per week until 18%, for a total of 5 weeks. While establishing the model, the following parameters were frequently monitored: blood pressure, TC, TG, HDL-c, LDL-c, AST and ALT. After 5 weeks of modelling, 36 rats were successfully modelled (SBP ≥140 mmHg) and divided into model group (MG, n = 9), valsartan group (VAL, 8 mg/kg, n = 9), BUDE low dose group (BUDE-L, 75 mg/kg, n = 9), BUDE high dose group (BUDE-H, 15 0 mg/kg, n = 9).

Following establishment of the model, all model animals continued to receive ACHSFD. Valsartan and BUDE groups were administered intragastrically the corresponding drugs daily for 12 consecutive weeks. Valsartan is a specific Angiotensin II Type 1 (AT1) receptor antagonist (Giles et al., 2017). Clinically, valsartan can not only reduce the blood pressure of hypertensive patients, patients with hypertension (Kintscher et al., 2010; Seferovic et al., 2017; Hanefeld and Abletshauser, 2001). Thus it is may improve metabolic hypertension.

### Blood Pressure Measurement

The BP-2010AUL small animal non-invasive pressure measurement system was used to measure blood pressures 2 h after drug administration. The specific operations were as follows: the temperature in the blood pressure room was established at 25 ± 1°C. A rat to be tested was introduced to the room and allowed to adapt to it for at least 15 min. The tail of the rat was warmed by enclosing the rat in a suitably sized squirrel cage equipped with a constant temperature blanket and adjusting the temperature to 40°C. The tail of the rat was put through a holder hole and fixed, and the system’s pulse sensor was inserted. When the signal wave stabilized, it automatically pressurized to measure and record systolic blood pressure (SBP), diastolic blood pressure (DBP) and mean arterial blood pressure (MBP).

### Biochemical Analyses

After 5 and 12 weeks of modelling, which included 7 weeks of BUDE administration, blood was taken from the ocular venous plexus. After 17 weeks of modelling, which included 12 weeks of BUDE administration, the rats were fasted overnight, and blood was collected from the abdominal aorta. Then the blood was bathed in a water bath at 37°C for 30 min, centrifuged twice at 3,500 rpm for 10 min each time to separate serum and plasma. TC, TG, HDL-c, LDL-c, AST and ALT in serum were detected by an automatic biochemical analyzer (HITACHI-7020, Japan) with corresponding kits.

### Enzyme-Linked Immunoassay

After 12 weeks of BUDE administration, blood was collected from the abdominal aorta into the anticoagulant centrifugal tube. Then the blood was bathed in a water bath at 37°C for 30 min, centrifuged twice at 3,500 rpm for 10 min each time to separate plasma. LPS, TNF-α, IL-6, D-LA, NO, ET-1 and eNOS in plasma were determined by enzyme-linked immunoassay with corresponding kits.

### Specimen Collection and Processing

Rats were fasted overnight with water and then anesthetized with pentobarbital. After blood collection from the abdominal aorta, the colon tissue was quickly removed and washed with normal saline at 4°C. The colon tissue near the cecum was cut into 1 mm^3^ spaced pieces and fixed in a 2.5% glutaraldehyde solution at 4 °C and protected from light. Colon tissue 2 cm away from the cecum was collected and quickly transferred to a cryotube, which was placed into liquid nitrogen for quick freezing and then stored at -80 °C. Samples of tissue from an area approximately 1 cm at the end of the colon were fixed in 4% paraformaldehyde solution for 24 h.

### Liver Index

Weighed the rat’s weight before anesthetized, removed the whole liver after blood collection from the abdominal aorta, and weighed the whole liver wet weight. Liver coefficient = liver wet weight (g)/body weight (g) × 100%.

### Histological Evaluations and Immunohistochemistry

The blood vessels and colon tissues fixed in 4% paraformaldehyde solution were removed, washed with tap water for 2 h, dehydrated with gradient ethanol and xylene, and then embedded in paraffin. The wax block was cut with a slicer with a thickness of 4 μm. Hematoxylin and eosin staining (H&E) was used to evaluate vessel and colon lesions in paraffin-embedded tissue sections. Immunohistochemistry (IHC) was used to stain the deparaffinized tissue. They were incubated with occludin, claudin-1 and TLR4 antibodies. Goat anti-rabbit or anti-mouse IgG was conjugated with HRP secondary antibody. After DAB staining, hematoxylin counterstained the nucleus, and the yellow one under the microscope was positive staining.

### Oil Red O Staining

After dissection, the liver tissue was removed and frozen at −80°C. The tissue was cut into 6 μm frozen sections, washed with distilled water for 30 s, soaked with 60% isopropanol for 30 s, stained with oil red O dye for 15 min. It was then placed in 60% isopropanol for 30 s and distilled water for 30 s, and the tissue was finally differentiated with hematoxylin and HCL.

### Scanning Electron Microscopy

The vessels of 1 mm^2^ were dissected and rinsed in precooled saline, then fixed overnight in 2.5% glutaraldehyde solution at 4°C. The ultrastructure of vessels was observed under field emission scanning electron microscope (SU8010, HITACHI, Japan) after rinsing, fixing with 1% osmium acid, dehydrated with gradient ethanol and tert-butanol, and dried in vacuum.

### Transmission Electron Microscopy

The colon tissues fixed in 2.5% glutaraldehyde solution were rinsed with PBS (pH7.0) for 3 times, and then fixed in 1% osmic acid solution. After rinsing three times with PBS, dehydrate with gradient ethanol and acetone, and then penetrate into graded epoxy resin and acetone with embedding agent. Finally, it was heated overnight at 70°C in a heating polymerizer. Ultrathin slices were cut into slices of 50–70 nm with an ultrathin microtome. The ultrastructure of the colon was observed by transmission electron microscopy (H-7650, HITACHI, Japan) at 20,000 times magnification.

### Western Blot Analysis

In short, the vessels tissues frozen at −80°C were removed and placed into in precooled RIPA buffer containing PMSF. After half an hour of lysis at 4°C, the vessel tissue homogenate was centrifuged at a speed of 12,000 rpm/min for 10 min, and the supernatants were collected. The total proteins concentration was detected by the BCA method, and then the protein sample was separated by 10% sodium dodecyl sulfate polyacrylamide gel electrophoresis (SDS-PAGE) and transferred to a polyvinylidene fluoride membrane. The proteins were used TLR4, MyD88, NF-κB p65, p-NF-κB p65, IκBα, p-IκBα, IKKβ and p-IKKβ antibody incubation at 4°C overnight. After that, the membrane was incubated in a suitable secondary antibody conjugated with horseradish peroxidase for 2 h and then washed three times in PBST solution. Finally, the ECL kit was used to display the protein bands, and the ImageJ software was used for analysis.

### QRT-PCR Analysis

The expressions of TLR4 and MyD88 were analyzed by qRT-PCR using gene-specific primers. Total RNA was extracted from vascular tissue according to the instructions of Trizol reagent (Invitrogen life technologies, Carlsbad, CA, United States), and the RNA concentration was measured by ultraviolet spectrophotometer. qRT-PCR was performed on the collected samples with a CFX384 touch real-time PCR detection system (Bio-Rad, Hercules, CA, United States). Rat-specific primers were designed by Sangon Biotech (Shanghai, China) Co., Ltd., and the detailed information was shown in Table 1. PCR amplification conditions were as follows: pre denaturation at 95°C for 1 min, and then 40 cycles of denaturation at 95°C for 15 s, annealing at 63°C for 25 s. The result was quantitatly analyzed by 2^−ΔΔCt [ΔCt=Ct (target)−Ct (GAPDH)]^.

**TABLE 1:** Real-time PCR primers.

Gene	Genbank accession	Primer Sequences (5’to3′)
Rat GAPDH	NM_017008.4	GAA​GGT​CGG​TGT​GAA​CGG​ATT​TG
		CAT​GTA​GAC​CAT​GTA​GTT​GAG​GTC​A
Rat MyD88	NM_198130.2	AGG​AGG​ACT​GCC​AGA​AAT​ACA​TAC
		GAT​GCC​TCC​CAG​TTC​CTT​TG
Rat TLR4	NM_019178	GAT​TGC​TCA​GAC​ATG​GCA​GTT​TC
		CAC​TCG​AGG​TAG​GTG​TTT​CTG​CTA​A

### Microbial Community Analysis by 16S rRNA Gene Sequencing Using Illumina Technology

The feces were collected by squeezing the abdomen of rats on the super clean table, stored in a high-pressure sterilized centrifuge tube, and stored at 80°C after quick freezing in liquid nitrogen. According to the European Soil Association soil DNA Kit (OMEGA Biotech Corp, Georgia, North Georgia), the microorganism DNA was extracted. The extraction quality of DNA was detected by 1% agarose gel electrophoresis. NanoDrop2000 UV-Vis spectrophotometer (Thermo Scientific, Wilmington, MA, United States) was used to determine the concentration and purity of DNA.

The hypervariable region of bacterial 16S rRNA gene V3-V4 was amplified with primer pairs 338F (5′-ACT​CCT​ACG​GGA​GGC​AGC​AG-3′) and 806R (5 ′-GGACTACHVGGGTWTCTAAT-3′) by an ABI GeneAmp^®^ 9700 PCR thermocycler (Applied Biosystems, CA, United States). The experiment was repeated 3 times for each sample. The product was purified with the AxyPrep DNA gel extraction kit (Axygen Biosciences, California, CA, United States) and quantified with Quantifluor™ Fluorometer (Promega, Madison, WI, United States).

DNA purified from a single sample was sequenced according to standard protocols by the Illumina Miseq platform (Illumina, San Diego, CA, United States). Operational taxonomic units (OTUs) with 97% similarity cutoff were clustered using UPARSE version 7.1, and chimeric sequences were identified and removed. The classification of each 16S rRNA gene sequence was analyzed using a 70% confidence threshold against Silva (SSU123) 16S rRNA database using the RDP classifier version 2.2. The scope of the data is the I-Sanger cloud platform (http://www.i-sanger.com) developed by Magi Biopharmaceutical Technology Co., Ltd. Results of the biochemical analysis were statistically compared using the Wilcoxon rank-sum test.

### Statistical Analysis

The results were analyzed using IBM SPSS version 22.0 and expressed as mean ± standard deviation (SD). Statistical significance was analyzed by one-way analysis of variance (ANOVA). When the homogeneity of variance was satisfied, the least significant difference *t*-test was applied. In addition, the Games-Howell method was used for analysis. The Spearman correlation coefficient was used to analyze the correlations between LPS, TNF-α, IL-6 and the relative abundance of the intestinal microbial community at the phylum and genus levels. All results were considered statistically significant at *p <* 0.05. Diagrams were prepared with GraphPad Prism version 8.

## Results

### BUDE Administration Lowered Blood Pressure in MH Rats

**FIGURE 2:**
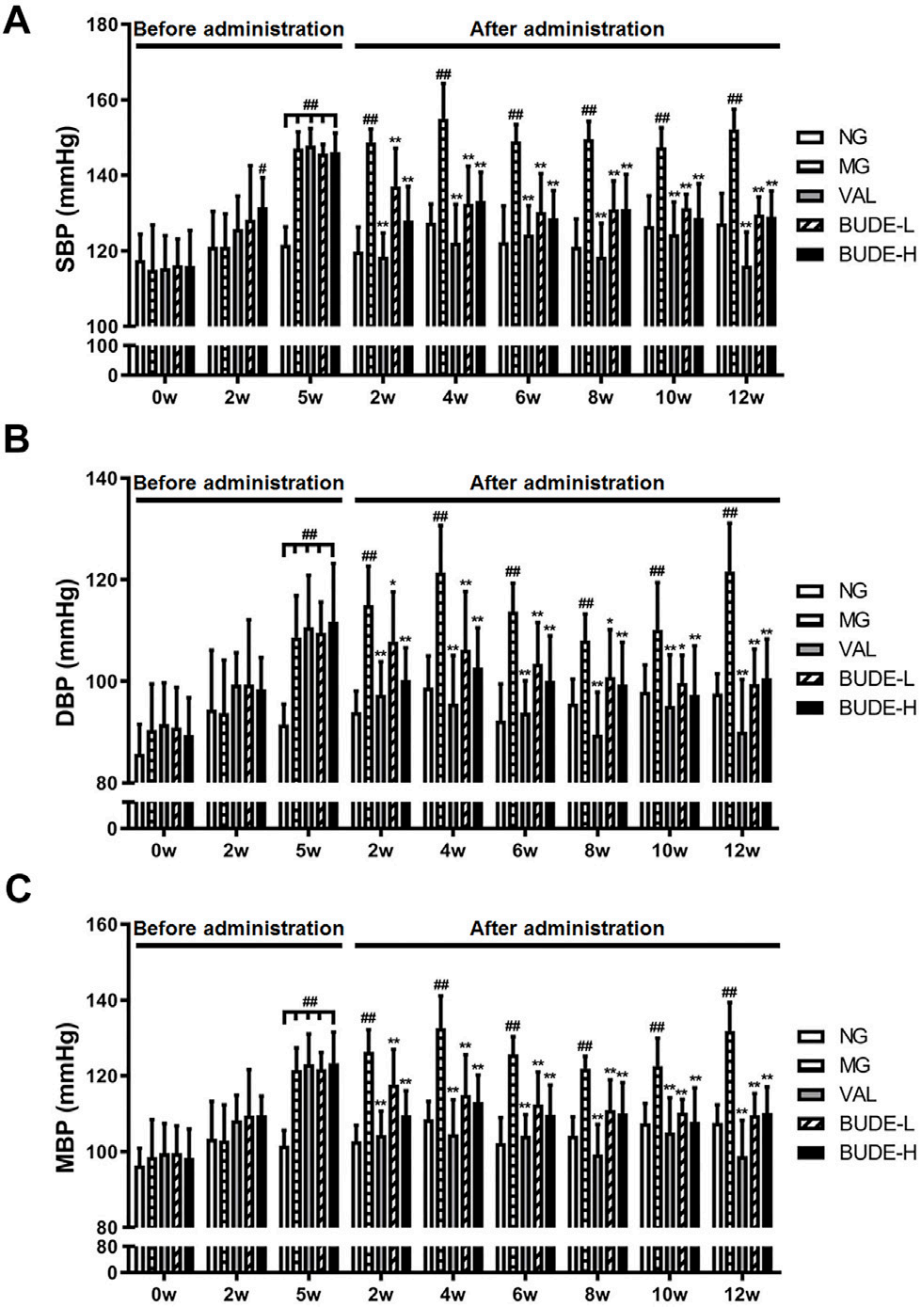
Effect of BUDE on the blood pressure of the model rats. **(A)** Changes in the systolic blood pressure (SBP). **(B)** Changes in the diastolic blood pressure (DBP). **(C)** Changes in the mean blood pressure (MBP). Compared with the normal control group ^#^ *p* < 0.05, ^##^ *p* < 0.01; compared with the model control group ^*^ *p* < 0.05, ^**^ *p* < 0.01, *n* = 8–10. NG: normal control group; MG: ACHSFDs model group; VAL: valsartan group; BUDE-L: BUDE low-dose group; BUDE-H: BUDE high-dose group.

At week 5, SBP, DBP and MBP of the rats in the MH model group were significantly higher than those of the control group (Figures 2A–C) (*p <* 0.01), and SBP was higher than 140 mmHg, indicating that the model was successfully established. After 2 weeks of administration, BUDE–H and BUDE–L significantly reduced the SBP, DBP and MBP of MH rats (Figures 2A–C) (*p <* 0.01, 0.05), and the efficacy was maintained and stabilized until 12 weeks of administration. Thus, these results demonstrate that ACHSFD administration significantly increases the blood pressure of rats and that administration of BUDE can lower the blood pressure of these MH model rats.

### BUDE Administration Reduced Blood Lipid and Serum Transaminase, and Improved Liver Pathological Injury in MH Rats

**FIGURE 3:**
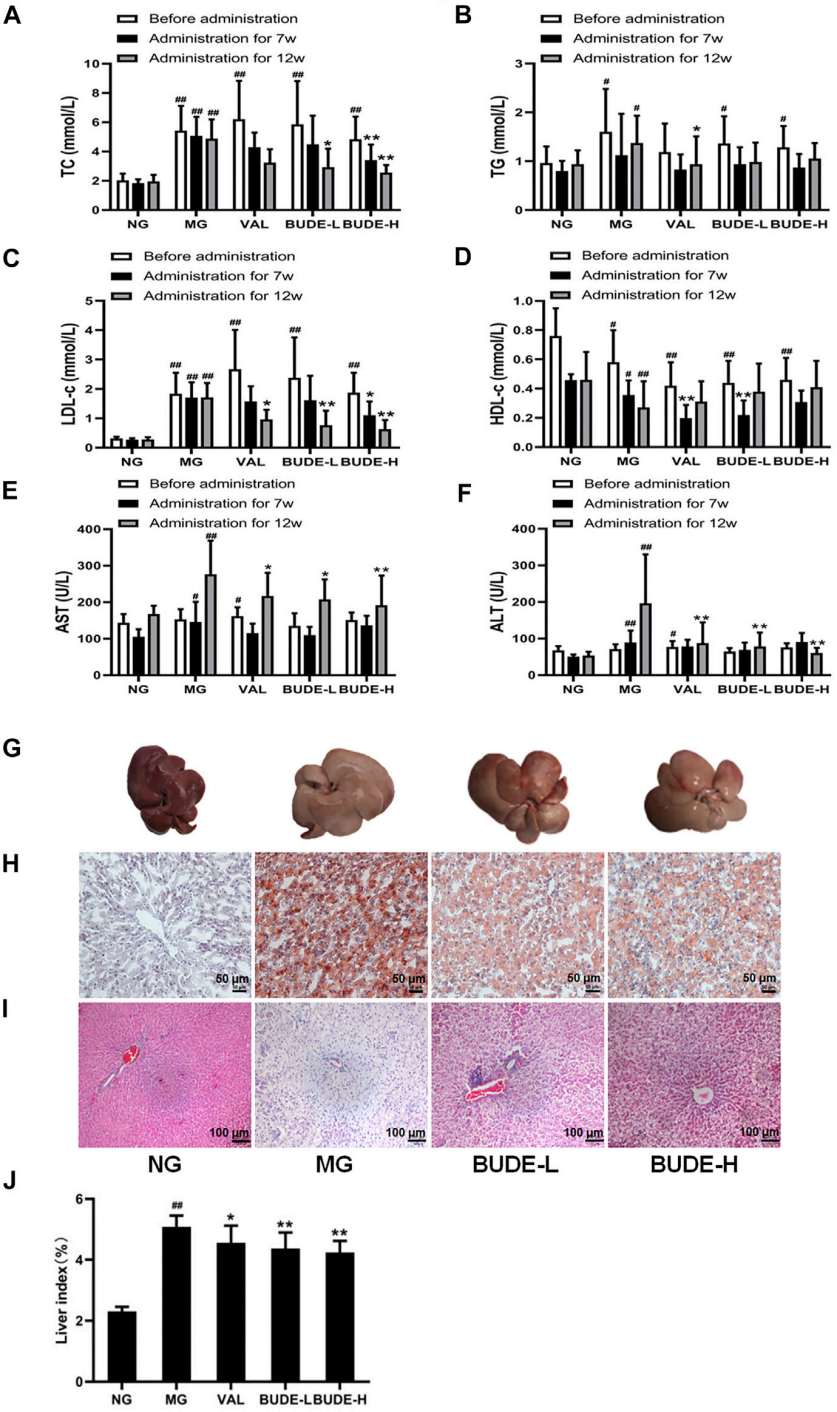
Changes of blood lipid, serum transaminase, and liver pathological. **(A)** Changes in the serum total cholesterol (TC). **(B)** Changes in the serum triglyceride (TG). **(C)** Changes in the serum low density lipoprotein cholesterol (LDL-c). **(D)** Changes in the serum high density lipoprotein cholesterol (HDL-c). **(E)** Changes in the serum aspartate aminotransferase (AST). **(F)** Changes in the serum alanine aminotransferase (ALT). **(G)** Liver gross morphology. **(H)** Oil red O staining of liver under magnification ×100 **(I)** Representative graph (×100) of hematoxylin and eosin staining of liver **(J)** Liver index. Compared with the normal control group ^#^ *p* < 0.05, ^##^ *p* < 0.01; compared with the model control group ^*^ *p* < 0.05, ^**^ *p* < 0.01, *n* = 7–10.

After 5 weeks of modelling, compared with the control group, the levels of serum TC, TG and LDL-C in the MH model group were increased (Figures 3A–C) (*p <* 0.01, 0.05), and the levels of HDL-c were decreased (Figure 3D) (*p <* 0.01, 0.05). After 12 weeks of modelling, which included 5 weeks of BUDE administration, compared with the control group, the levels of AST and ALT in the MG were significantly increased (Figures 3E,F) (*p <* 0.01, 0.05).

After 7 weeks of administration, BUDE-H could decrease the TC and LDL-C (*p <* 0.01, 0.05). After 12 weeks of administration, BUDE-H and BUDE-L could significantly decrease AST, ALT, TC and LDL-C levels in serum of model rats (*p <* 0.01, 0.05), while TG and HDL-C levels showed a trend of improvement, but there was no significant difference.

The results of H&E and oil red O also showed liver dyslipidemia and liver injury in MH rats. We noticed that the liver tissue of rats in the control group was dark red in color with smooth and shiny surface and soft texture. In contrast, the general shape of the liver of rats in the MH model group changed dramatically. The liver volume was significantly increased, with whiten and greasy surface and slightly harder texture (Figure 3G), and the liver index was significantly increased (Figure 3J) (*p <* 0.01). Compared with the control group, BUDE-H improved the liver morphology and decreased the liver index by 16.47%. Then, H&E staining showed extensive hepatocyte balloon-like lesions in the liver of model rats (Figure 3I). Oil red O staining showed abnormal lipid accumulation in liver of rats in the MH model group (Figure 3H). And BUDE-H significantly reduced these lesions. The results showed that BUDE had a certain improvement effect on the abnormal lipid metabolism and liver pathological injury. It was indicated that BUDE was beneficial to the prevention and treatment of MH.

### BUDE Administration Improved Vascular Endothelial Function in MH Rats

**FIGURE 4:**
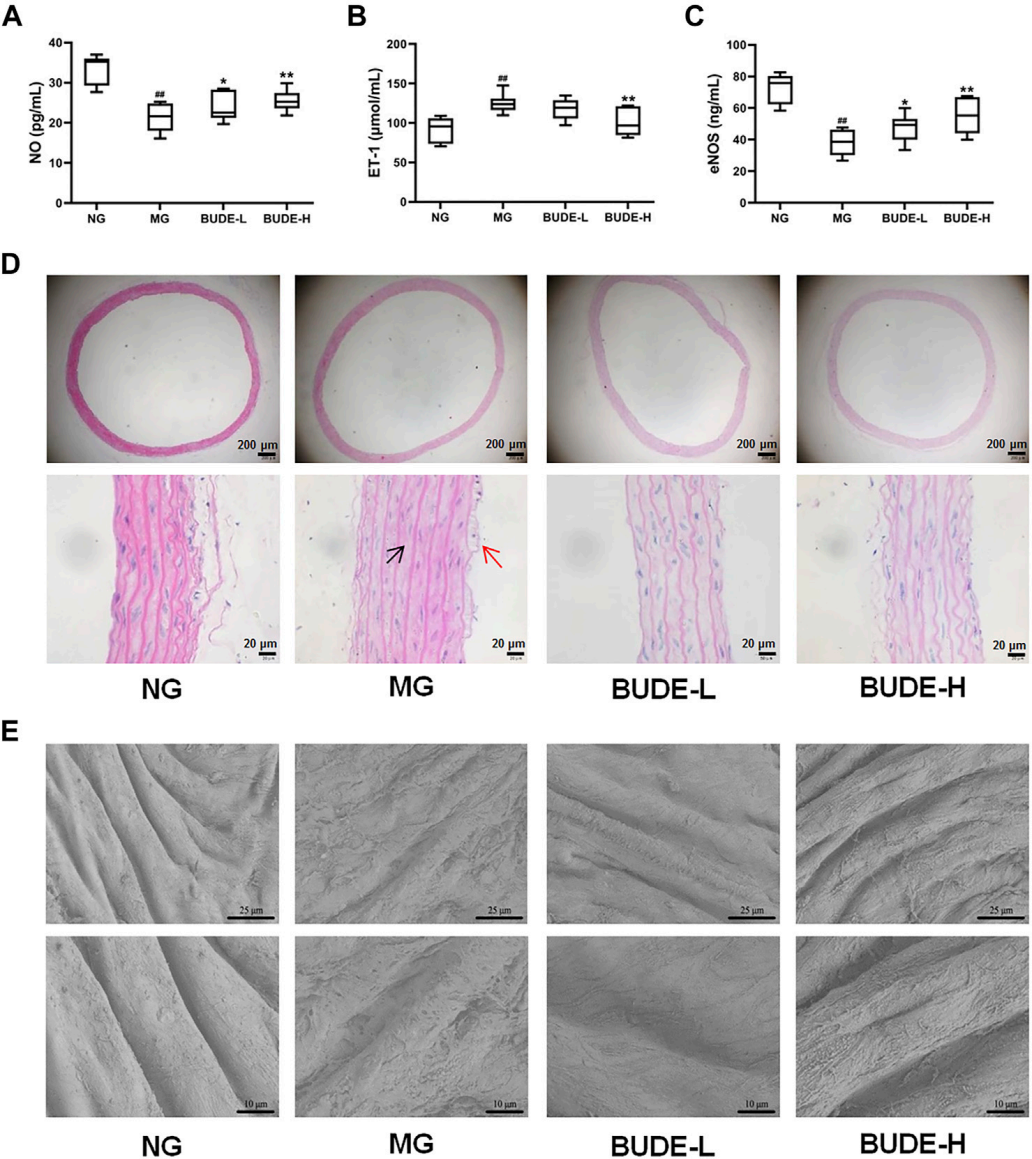
Effect of BUDE on vascular endothelial. **(A)** Changes in the plasma nitric oxide (NO). **(B)** Changes in the plasma endothelin 1 (ET-1). **(C)** Changes in the plasma endothelial nitric oxide synthase (eNOS). **(D)** Representative graph ( × 40, × 400) of H&E staining of the vessels: shedding of endothelial cells (red arrow) and smooth muscle cell hypertrophy (black arrow). **(E)** Vessel ultrastructure by scanning electron microscopy (SEM) observed at magnification 1,000× and 2000×. Compared with the normal control group ^#^ *p* < 0.05, ^##^ *p* < 0.01; compared with the model control group ^*^ *p* < 0.05, ^**^ *p* < 0.01, *n* = 9.

Compared with the control group, the plasma levels of NO and eNOS in the MH model group were significantly reduced (Figures 4A–C) (*p <* 0.01), and the plasma levels of ET-1 were significantly increased (Figure 4B) (*p <* 0.01). These changes are consistent with damage to the vascular endothelium. Administration of BUDE-L and BUDE-H significantly increased the plasma levels of NO and eNOS (*p <* 0.01, 0.05) and decreased the plasma levels of ET-1 (*p <* 0.01) of the model MH rats.

The results of H&E staining showed that compared with the control group, the vascular endothelium was disrupted, and the smooth muscle cells proliferated in the model group (Figure 4D). However, BUDE alleviated these injuries in model rats. In further observing the damage of the vascular endothelial structure, we found by scanning electron microscopy that the vascular endothelial structure of control rats was complete and smooth, distributed in a parallel shape with obvious boundaries and no cell adhesion, while the vascular endothelial structure of model rats had a rough surface, blurred boundaries and abundant cell adhesion (Figure 4E). As shown in Figure 4E, BUDE administration improved the vascular endothelial structure abnormalities seen in the model rats.

### BUDE Administration Attenuated Activation of the TLR/MyD88 Pathway in the Vasculature of MH Rats

**FIGURE 5:**
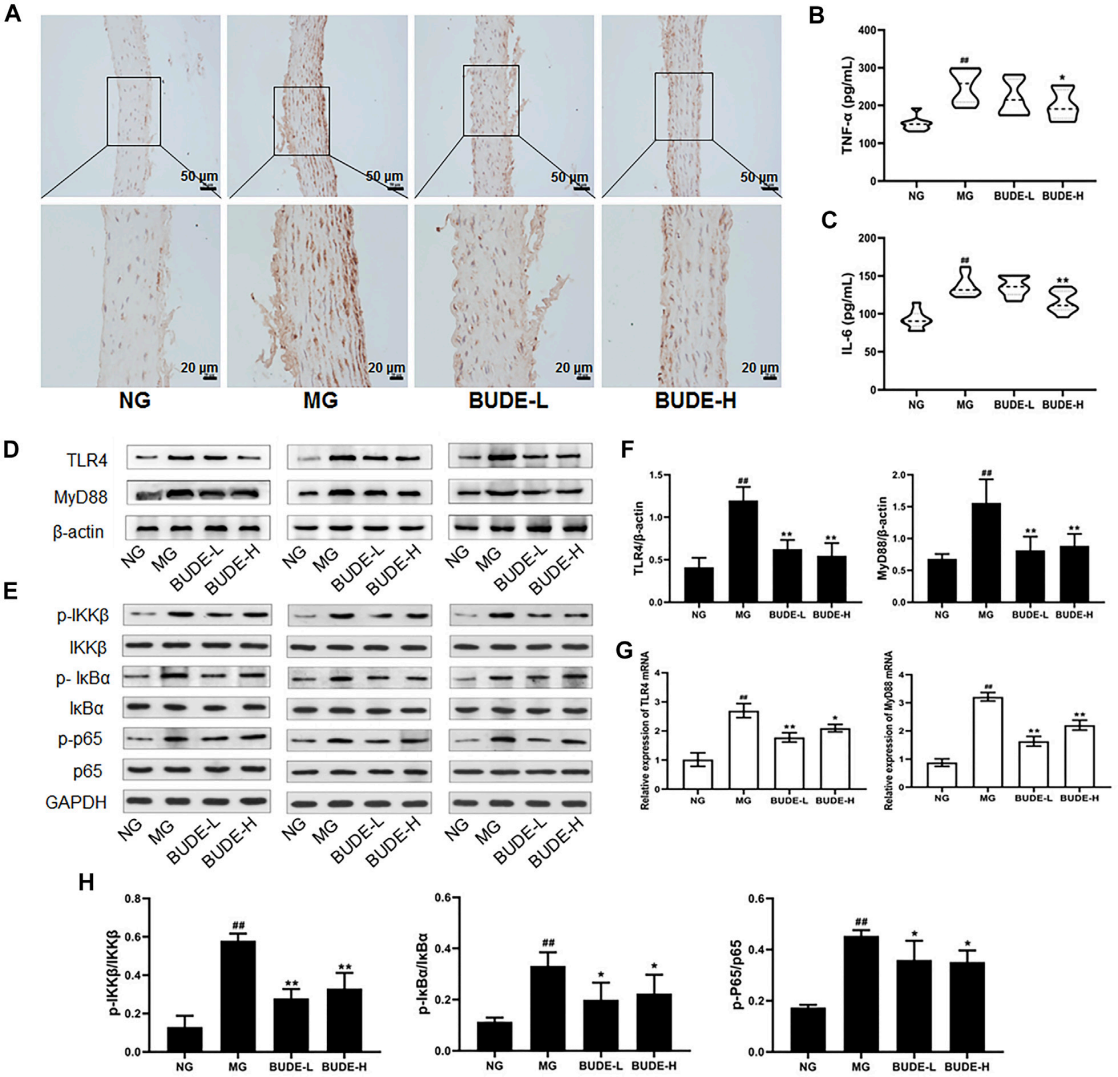
Effect of BUDE on the TLR4/MyD88 pathway in the vessel. **(A)** Immunohistochemical staining of TLR4 in the vessel (100×, 200×). **(B)** Changes in the plasma tumor necrosis factor-α (TNF-α) **(C)** Changes in the plasma interleukin-6 (IL-6). **(D-F, H)** Western blot assaying on the levels of TLR4, MyD88, IKKβ and phosphorylation, IKBα and phosphorylation, p65 and phosphorylation in the vessel **(G)** Relative expressions of TLR4 and MyD88 mRNA. Compared with the normal control group ^#^ *p* < 0.05, ^##^ *p* < 0.01; compared with the model control group ^*^ *p* < 0.05, ^**^ *p* < 0.01, *n* = 3, 9.

Compared with control rats, the plasma levels of TNF-α and IL-6 in the MH model rats were significantly increased (*p <* 0.01), and the plasma levels of TNF-α and IL-6 in model rats were decreased by BUDE-H administration (Figures 5B,C) (*p <* 0.01, 0.05). The results of immunohistochemistry, Western blotting, and qPCR showed that the expression of TLR4 and MyD88 proteins and mRNA in the vessels of model rats were significantly increased compared with those in the control group (*p <* 0.01), and these expression levels were down-regulated by administration of BUDE (Figures 5A,D,F,G) (*p <* 0.01, 0.05).

In a further experiment, Western blot analyses demonstrated that the phosphorylation levels of IKKβ, IκBα and NF-κB p65 were significantly increased, and BUDE administration reduced these phosphorylation levels (Figures 5E,H) (*p <* 0.01, 0.05). These results are consistent with a model in which BUDE reduces the release of inflammatory cytokines through inhibition of the TLR4/MyD88 signaling pathway.

### BUDE Administration Limited Disorders in Intestinal Microbiota in MH Rats

**FIGURE 6:**
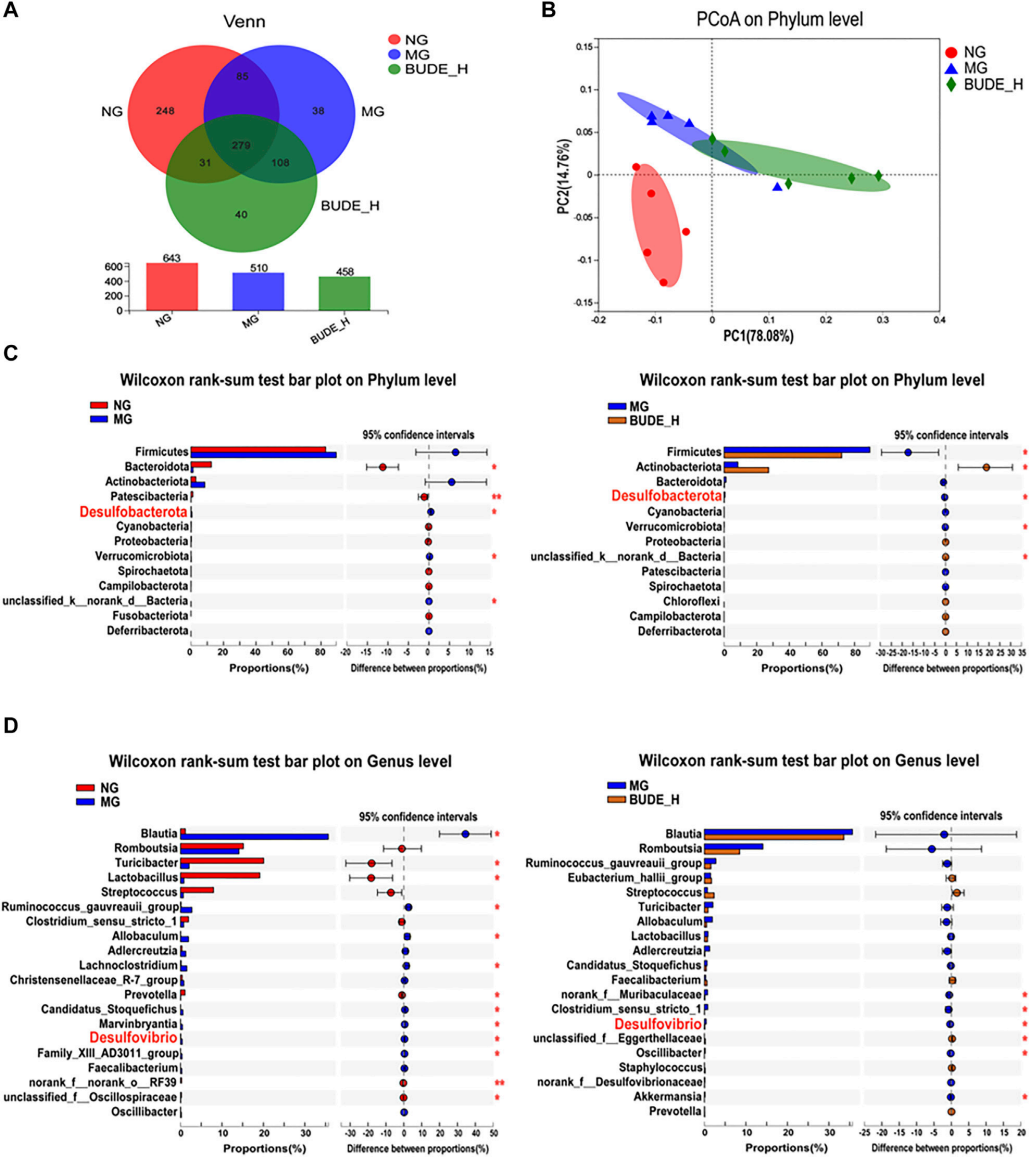
Effect of BUDE on the intestinal flora. **(A)** Venn diagram. **(B)** PCoA analysis of phylum level in groups. **(C)** Analysis of the difference microbiota abundance between the NG, MG and BUDE-H at the phylum level. **(D)** Analysis of the difference of the top 50 of intestinal flora abundance between the NG, MG and BUDE-H groups at the genus level. ^*^ *p* < 0.05, ^**^ *p* < 0.01, *n* = 5.

Through Venn diagram analyses, the number of OTU samples from MH model rat intestinal tracts was found to be reduced relative to the control, but there were no notable differences with other groups (Figure 6A). A clear separation was observed in the PCoA between the control, model and BUDE-H groups (Figure 6B), indicating the gut environments were extremely different between three groups. Thus, compared with the control group, the composition of intestinal microflora in the model group changed significantly. Instead of restoring the community structure, BUDE-H administration thus might reshape the composition of the flora.

To assess specific changes in the intestinal microbiota that were closely associated with LPS production, we compared the relative abundances of the major taxa identified by sequencing. At the phylum level (Figure 6C), compared with the control group, the relative abundances of species of *Desulfurobacterota* and *Verrucomicrobiota* were significantly increased in the model group, and the relative abundances of *Bacteroidota* and *Patescibacteria* species were significantly decreased (*p <* 0.01, 0.05). Compared with untreated model rats, the relative abundances of *Desulfurobacterota*, *Verrucomicrobiota* and *Firmicutes* were reduced in model rats treated with BUDE-H (*p <* 0.05). At the genus level (Figure 6D), compared with the control group, the relative abundances of *Ruminococcus gauvreauii* and species of *Blautia*, *Allobaculum*, *Lachnoclostridum*, and *Desulfovibrio* were increased in the model group, and the relative abundances of *Turicibacter*, *Lactobacillus* and *Prevotella* were decreased. Compared with untreated model rats, model rats treated with BUDE-H had reduced abundances of *Desulfovibrio*, *norank_f_Muribaculaceae*, and *Oscillibacter* (*p <* 0.05).

**FIGURE 7:**
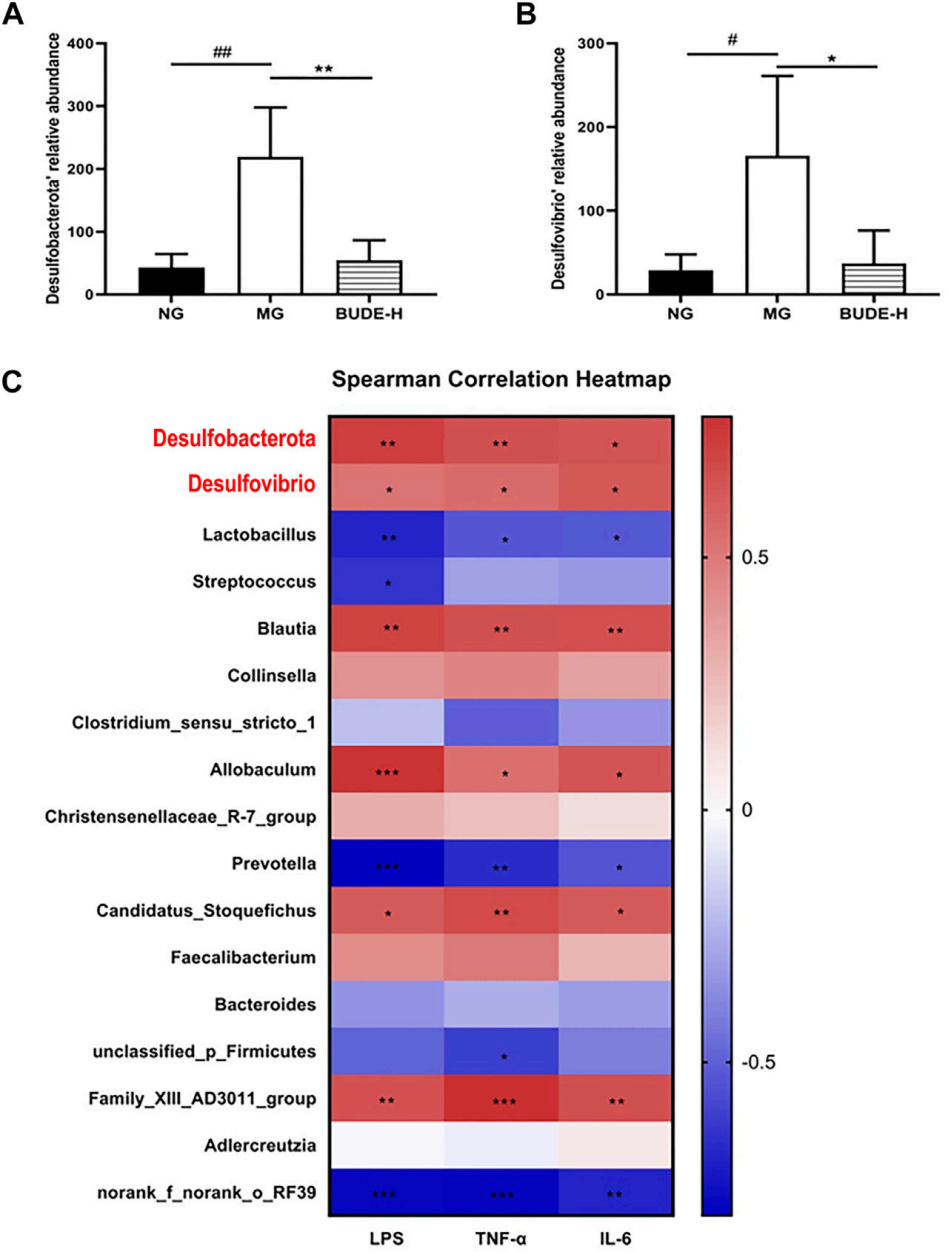
Correlation analysis between LPS and intestinal flora. **(A)** Relative abundance of *Desulfobacterota*. **(B)** Relative abundance of *Desulfovibrio*. **(C)** The spearman correlation between LPS, TNF-α, IL-6 and intestinal flora. Compared with the normal control group ^#^ *p* < 0.05, ^##^ *p* < 0.01; compared with the model control group ^*^ *p* < 0.05, ^**^ *p* < 0.01, *n* = 5.

*Desulfovibrio* and *Desulfurobacterota* are Gram-negative bacteria thus contain LPS as a major part of their cell walls. We found that *Desulfobacterota* and *Desulfovibrio* are main bacteria in rats of all groups analyzed but that BUDE-H administration significantly reduced their relative abundance in model rats (Figures 7A,B) (*p <* 0.01, 0.05). Correlations between LPS, TNF-α, IL-6 and the relative abundances of the gut microbial community were analyzed by Spearman’s correlation coefficient heat maps, which showed that, as expected, *Blautia*, *Desulfobacterota* and *Desulfovibrio* correlated positively with LPS, TNF-α, and IL-6 (Figure 7C) and that *Lactobacillus* showed a negative correlation with the levels of these chemicals.

### BUDE Administration Alleviated Intestinal Barrier Damage in MH Rats

**FIGURE 8:**
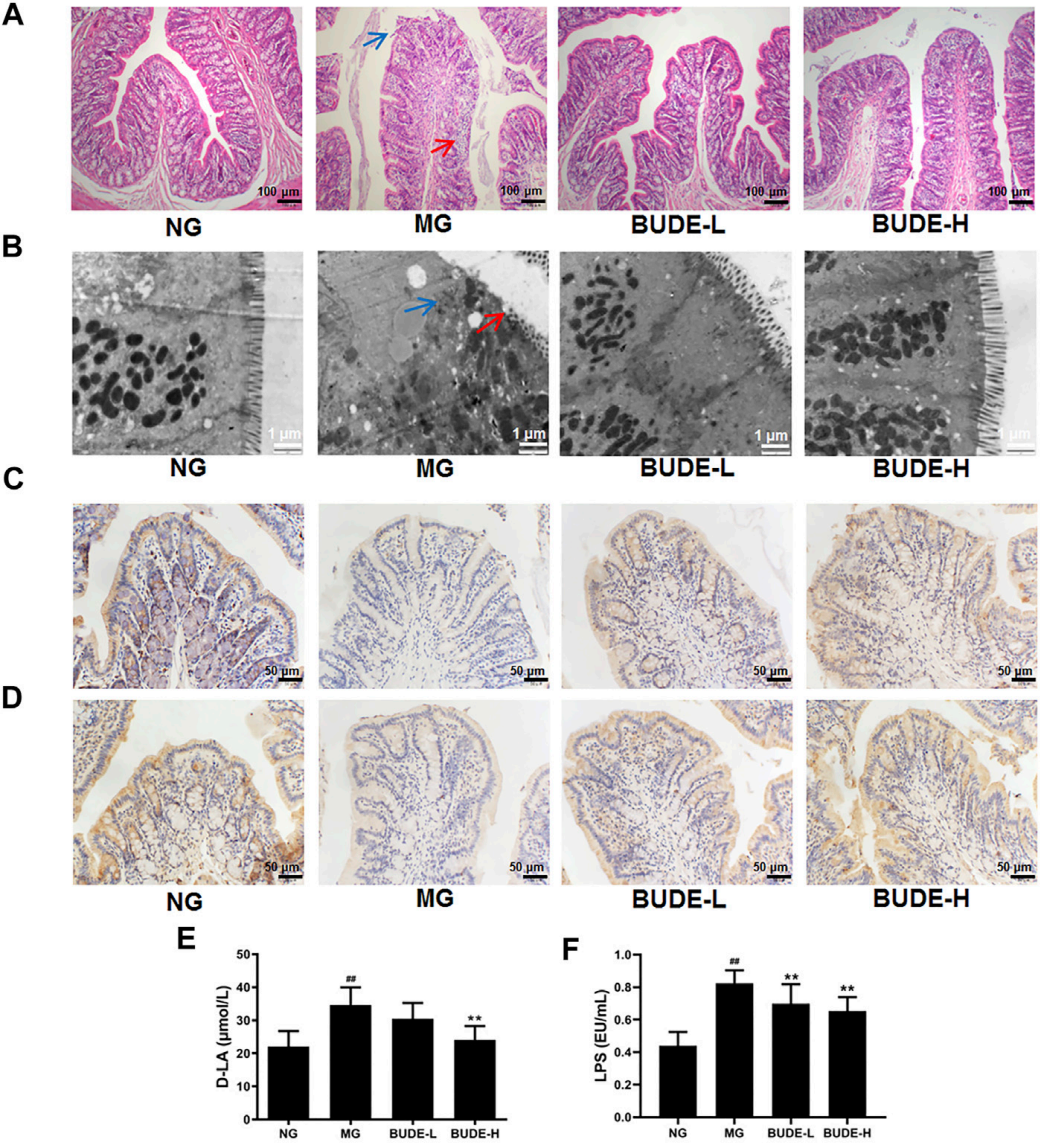
Effect of BUDE on intestinal barrier. **(A)** Representative graph (×200) of hematoxylin and eosin staining of the colon: shedding of colonic villi (blue arrow) and a small amount of inflammatory cell infiltration (red arrow) **(B)** Colon ultrastructure by transmission electron microscopy (TEM) observed at magnification × 20000: Villi loss (red arrow) and tight junction protein damage (blue arrow). **(C)** Representative figures of immunohistochemical staining of claudin-1 in the colon (×200). **(D)** Representative charts of immunohistochemical staining of occludin in the colon (×200). **(E)** Changes in the plasma d-lactic acid (D-LA). **(F)** Changes in the plasma lipopolysaccharide (LPS). Compared with the normal control group ^#^ *p* < 0.05, ^##^ *p* < 0.01; compared with the model control group ^*^ *p* < 0.05, ^**^ *p* < 0.01, *n* = 3, 9.

The results of H&E staining showed that compared with the control group, colonic villi of model rats were shed and exhibited mild inflammatory cell infiltration (Figure 8A). To further investigate these changes, the ultrastructure of the large intestine was observed by transmission electron microscopy. We found that villi in the model group were short and defective, and tight junctions were destroyed (Figure 8B). Compared with the untreated model group, BUDE was found to alleviate this pathological injury of the colon.

Immunohistochemistry showed that the expression levels of occludin and claudin-1 in the colon of model rats were decreased, and these levels were increased after administration of BUDE-H and BUDE-L (Figures 8C,D). The results of an enzyme immunoassay showed that the content of D-LA in the plasma of rats in the model group was higher than that of the control group, indicating that the intestinal permeability of the model rats was increased. The content of D-LA in plasma was decreased by BUDE administration (Figure 8E). Moreover, the plasma level of LPS in the model group was significantly increased compared with controls (*p <* 0.01), and BUDE administration reduced the plasma level of LPS in model rats (Figure 8F) (*p <* 0.01). These results suggest that ACHSFD causes intestinal barrier damage, which may lead to increased LPS penetration into the blood, and that BUDE can alleviate intestinal barrier damage and reduce plasma LPS content.

## Discussion

MH is characterized by various metabolic disorders and low-grade inflammation, and it frequently leads to cardiovascular and cerebrovascular complications. The etiology and mechanism of its formation are complicated. Zhu et al. suggested that an unhealthy diet is the initial culprit (Zhu et al., 2013). Thus, we established an MH model by induction with ACHSFD, which appears to simulate the unhealthy diets in many humans (Chen et al., 2015; Li et al., 2021). We found that several phenomena in the MH rats were similar to those in human MH patients, including high blood pressure and increasing of serum lipid levels and markers of liver damage. These changes indicated that elevated blood pressure in MH rats was accompanied by abnormal lipid metabolism and changes to liver function.

To attempt to address these changes, we turned to *Chrysanthemum indicum* L., which is a traditional Chinese herb commonly used for the treatment of hypertension. Its history can be traced back to the Ming Dynasty (Tseng et al., 2020). Flavonoids, mainly composed of BUD, are the most important antihypertensive active components in *Chrysanthemum indicum* L. (He et al., 2012), and flavonoids have been shown to exhibit antimicrobial and anti-inflammatory pharmacological activities (Cheng et al., 2005; Zhu et al., 2005). Our team had previously isolated and purified BUDE, and found that BUDE could lower the blood pressure and improve the liver and kidney functions of spontaneously hypertensive rats (Yin et al., 2014). In the present work, it was found that BUDE at 75 or 150 mg kg^−1^ reduced the blood pressure of MH rats after 2 weeks of treatment, and efficacy was maintained until 12 weeks of treatment. At week seven of administration, BUDE-H significantly reduced serum TC and LDL-C levels, and at week 12 of administration, both BUDE-L and BUDE-H decreased serum TC, LDL-C, AST and ALT levels. In addition, administration decreased the liver index of MH rats and improved pathological injury, including vacuolation of hepatic cells vacuolation and lipid deposition. These results suggested that BUDE was of benefit to the treatment of MH.

The pathogenesis of MH is complex; the gastrointestinal tract is considered to be the initial organ of onset (Li et al., 2021), and intestinal LPS is the key factor driving MH. LPS is released from cell walls of dead Gram-negative bacteria such as *Desulfovibrio* (Qureshi et al., 1999) and is cleared by the liver (Szabo and Bala. 2010). When LPS production is increased or the intestinal barrier is disrupted, it goes beyond the liver’s handling capacity, resulting in an increase in circulating plasma LPS, and stimulating the release of inflammatory cytokines such as TNF-α and IL-6, thereby causing chronic low-grade inflammation of vessels, liver and other organs throughout the body, thus inducing MH. In this study, we found that plasma LPS, TNF-α and IL-6 levels were significantly increased in rats with MH as induced by ACHSFD, which is consistent with the work of other researchers in both patients and animals (Raybould, 2012; Kim et al., 2018; Lei et al., 2019). We also found that BUDE can reduce the plasma levels of LPS, TNF-α and IL-6 in MH rats. Therefore, BUDE may improve inflammation by decreasing LPS levels.

After LPS penetrates the intestinal barrier and enters the systemic circulation, it further induces the activation of the TLR4/MyD88 pathway in blood vessels, the liver, and other organs. TLR4 is a receptor for LPS and is highly expressed on vascular endothelial cells (De Batista et al., 2014), and its activation on endothelial cells activates MyD88. Subsequently, the IKK complex is stimulated to phosphorylate IκBα, which leads to the degradation of IκBα, exposing the nuclear localization site of NF-κB, and leading to the rapid transfer of free NF-κB to the nucleus (Zandi et al., 1997). Nuclear NF-κB induces the expression of inflammatory factors, which further causes vascular endothelial injury, vasomotor factor imbalance, and other pathologic issues (Kang et al., 2009; Kayagaki et al., 2013; Su et al., 2021), eventually disrupting vascular function and increasing blood pressure. The immunohistochemistry results of this study showed that the expression of TLR4 protein in the vasculature of MH rats increased. Further Western blot analyses showed that the expression of TLR4 and MyD88 proteins and mRNA in the vasculature of MH rats was significantly upregulated. In addition, the phosphorylation level of IKBα, IKKβ and NF-κB p65 was elevated. These results suggest that LPS released upon the generation of the MH model might activate the TLR4 pathway, leading inflammation and systemic damage.

Flavonoids such as luteolin and naringenin have been reported to inhibit TLR4/NF-κB signal transduction (Katherine et al., 2016). Our team found that BUD can inhibit vascular inflammatory damage induced *in vitro* and can decrease the expression of NF-κB related proteins in vascular cells (Xu et al., 2016). In the present study, it was found that BUDE could not only decrease the plasma LPS content, but it could also down-regulate the expression of TLR4 and MyD88 proteins and mRNA in the blood vessels of MH rats and inhibit the phosphorylation of IKKβ, IκBα and NF-κB p65. Thus, these findings are consistent with our previous results. Therefore, we speculate that BUDE might delay the process of MH by inhibiting the enteric-origin LPS/TLR4 signaling pathway.

Vascular endothelial injury has been shown to be closely related to endothelial dysfunction (Bautista, 2003), which leads to hypertension. NO and ET-1 are important substances produced by vascular endothelial cells to regulate the vasomotor function of vascular smooth muscle cells (VSMC) (Li et al., 2020). Normally, NO and ET-1 are in dynamic equilibrium, and VSMC shows a well-differentiated contractile phenotype (Owens et al., 2004). When vascular injury leads to endothelial dysfunction, NO synthesis decreases and ET-1 secretion increases, and this contributes to hypertension (Monica ET al., 2016; Wong et al., 2013). Our team previously found vascular endothelial injury in MH rats, and the expression of eNOS protein in the vessel and the NO level in the serum were significantly decreased (Li et al., 2021). Here, the plasma levels of eNOS and NO in MH rats were decreased, and the ET-1 level was increased, which was consistent with the previous results. BUDE increased the plasma eNOS level of MH rats and corrected the imbalance of NO/ET-1 levels of vasoactive substances. When we further studied the effects of BUDE on vascular function and structure, we found that BUDE could improve the morphological changes of the vasculature (endothelial cell shedding, smooth muscle cell hypertrophy) and endothelial ultrastructure abnormalities (severe endothelial cell shedding, rough surface and cell adhesion) in MH rats.

ENOS participates in NO synthesis. Nunes et al. (2017) found that blocking TLR4 function with an anti-TLR4 antibody and other treatments increased NO production, and Zhang et al. (2018) found that overexpressed MyD88 down-regulated eNOS through the TLR4/MyD88 signaling pathway. Similarly, in another study, endothelial NO levels in eNOS, TLR4 and MyD88 knockout mice were examined, and TLR4 activation was found to reduce the expression of eNOS in endothelial cells through the MyD88 pathway (Yazji et al., 2013). These results indicated that the TLR4/MyD88 signaling pathway has an important relationship with NO. Combined with the above results, we speculated that BUDE might reduce vascular endothelial injury by inhibiting the TLR4/MyD88-related pathway, thus improving vasodilation function and lowering blood pressure.

The level of LPS in plasma is closely related to the intestinal micro ecological balance and intestinal mucosal barrier. Intestinal microorganisms are the source of LPS and are closely related to hypertension (Richards et al., 2017). Gut microbes are a key source of LPS, and diet is a critical factor affecting the composition of the gut microbiome (Marques et al., 2018). For example, a 4 weeks high-fat diet significantly increased plasma LPS levels by 2–3-fold (Katherine et al., 2016). Long-term ACHSFD administration leads to intestinal microbial dysregulation (Li et al., 2020); for instance, the relative abundance of Gram-negative *Desulfovibrio* has been shown to increase with ACHSFD (Lei et al., 2019). However, dietary flavonoids like quercetin attenuate the intestinal dysbiosis induced by high-fat diet (Etxeberria et al., 2015; Wang JH. et al, 2018).

The results of this study showed that the abundance of flora was changed in rats. At the phylum level, the abundance of *Desulfobacterota* was increased in the model rats, and BUDE was found to reduce the abundance of *Desulfobacterota* under these conditions. At the genus level, the abundances of *Blautia*, *Ruminococcus*, *Allobaculum*, *Lachnoclostridum*, and *Desulfovibrio* were increased, while *Turicibacter*, *Lactobacillus* and *Prevotella* were decreased in the model. BUDE was found to reduce the abundance of *Desulfovibrio*, *norank_f_Muribaculaceae*, and *Oscillibacter*. We found that *Desulfobacterota* and *Desulfovibrio* are the main differential bacteria between the control group, the model group and the BUDE-H group. They are sulfate-reducing bacteria, producing the sulfide to thin the mucin layer, thus increasing intestinal permeability. Moreover, further analyses showed that *Desulfobacterota* and *Desulfovibrio* abundances are positively correlated with levels of LPS.

How does consumption of high-sugar, high-fat, and alcohol contribute to increases in blood LPS? One possibility is raised intestinal permeability, with LPS crossing the intestinal barrier more rapidly. This study found that BUDE could up-regulate the expression of claudin-1 and occludin proteins in the colons of MH rats, and reduce the level of plasma D-LA. Further examination of colon morphology and ultrastructure showed that BUDE could improve the pathological damage of villi shedding in colon tissue of MH rats. These results suggest that, in addition to effecting changes to the microbiome, the improvement of the intestinal barrier is another critical mechanism through which BUDE reduced the LPS level in the plasma of MH rats.

This hypothesis is supported by the following studies. The integrity of tight junctions in the intestinal epithelium of mice on long-term high fat, fructose and alcohol has been shown to be impaired, and elevated LPS has been associated with leakage (Katherine et al., 2016; Salazar et al., 2020; Jamar et al., 2021). Clinically, the expression of tight junction proteins, such as occludin and claudin-1, was found to be decreased in the colons of alcohol-dependent subjects (Cho and Song, 2018). In addition, the increase of LPS level in intestinal tissue (Guo et al., 2015) and the relative abundance of *Desulfovibrio* (Kushkevych et al., 2019; Lei et al., 2019) accelerate intestinal barrier damage, which increases intestinal permeability. These changes allow LPS to enter the systemic circulation through the intestinal barrier, and this seems to be associated with increased plasma LPS levels in hypertensive patients and animals fed with a high-fat diet (Kim et al., 2012; Katherine et al., 2016). Interestingly, flavonoids such as naringenin, luteolin and puerarin can improve intestinal barrier dysfunction (Katherine et al., 2016; Murota et al., 2018).

**FIGURE 9:**
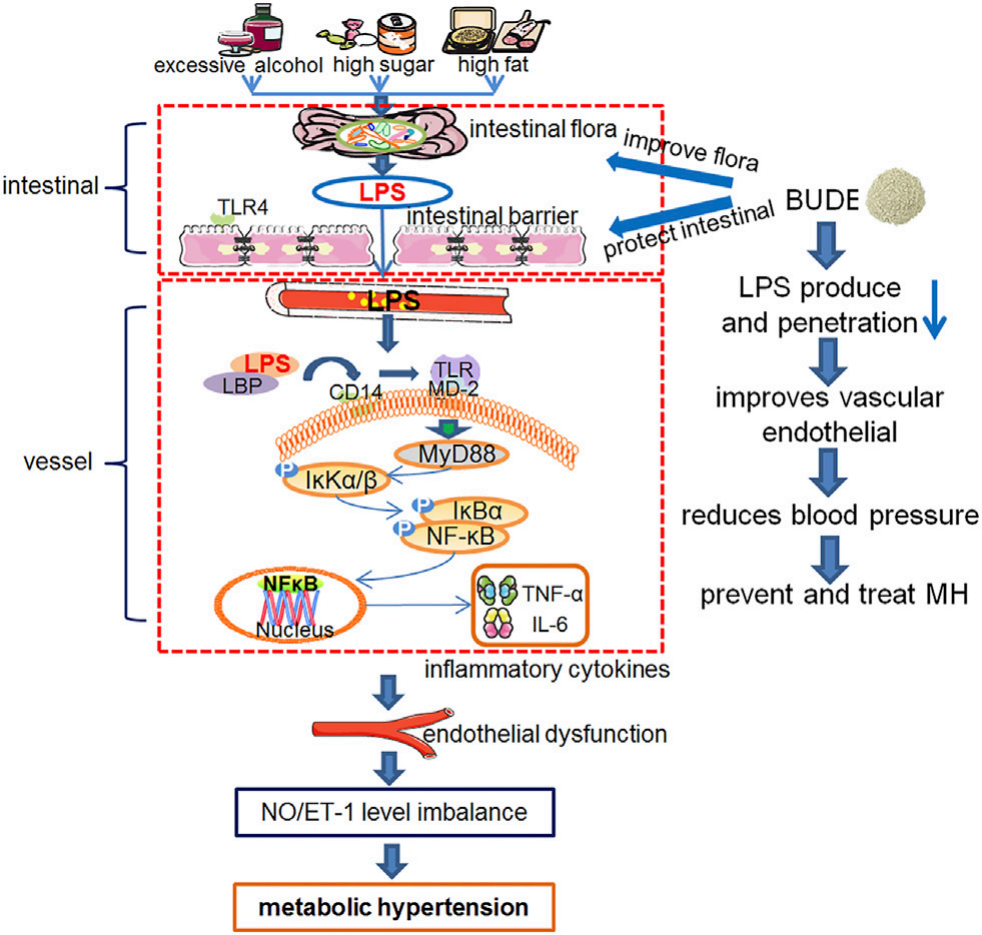
Experiment protocol blueprints. This schematic diagram shows the mechanism of over-consumption of alcohol, and high sugar and fat diets (ACHSFDs) leading to metabolic hypertension (MH). BUDE regulated intestinal flora and improved the intestinal barrier, so as to reduce LPS production and infiltration into the blood circulation, inhibited the activation of the enteric-origin LPS/TLR4 pathway, and improved vascular endothelial function, thereby improving MH.

BUDE was found to improve intestinal flora, reduce the relative abundance of *Desulfovibrio* and *Desulfurobacterota*, and improve intestinal barrier damage. In addition, it can inhibit TLR4/MyD88 pathway by reducing LPS invasion into vessels, and lower blood pressure by improving vasomotor function. Moreover, it can reduce the blood lipid level of MH rats and improve liver pathological damage and lipid deposition. Therefore, BUDE has great potential in the prevention and treatment of MH. An overview of the experiments conducted in this research is presented in Figure 9.

## Conclusion

BUDE regulated intestinal flora and improved the intestinal barrier. Thus, BUDE reduced LPS production and infiltration into the blood circulation, inhibited the activation of the enteric-origin LPS/TLR4 pathway, and improved vascular endothelial function, thereby lowering blood pressure in MH rats. This research proposed a new mechanism of the activity of BUDE against MH through the inhibition of the enteric-origin LPS/TLR4 pathway.

## Data Availability

The datasets presented in this study can be found in online repositories. The names of the repository/repositories and accession number(s) can be found below:NCBI Sequence Read Archive (SRA) database under the Accession Number of SRP337707.

## Abbreviations

MH, metabolic hypertension; BUD, buddleoside; BUDE, buddleoside-rich Chrysanthemum indicum L. extract; TLR4, Toll-like receptor 4; LPS, lipopolysaccharide; ACHSFDs, over-consumption of alcohol, and high sugar and fat diets; TNF, tumor necrosis factor; IL, interleukin; TC, total cholesterol; TG, triglyceride; LDL-c, low density lipoprotein cholesterol; HDL-c, high density lipoprotein cholesterol; ALT, alanine aminotransferase; AST, aspartate aminotransferase; NO, Nitric oxide; ET-1, endothelin 1; eNOS, endothelial nitric oxide synthase; D-LA, D-lactic acid; SHR, spontaneously hypertensive rats; SBP, systolic blood pressure; DBP, diastolic blood pressure; MBP, mean arterial blood pressure; H&E, hematoxylin and eosin; IHC, immunohistochemistry; SEM, scanning electron microscopy; TEM, transmission electron microscopy.
